# Mechanisms of High Temperature Resistance of *Synechocystis* sp. PCC 6803: An Impact of Histidine Kinase 34

**DOI:** 10.3390/life5010676

**Published:** 2015-03-02

**Authors:** Jan Červený, Maria A. Sinetova, Tomáš Zavřel, Dmitry A. Los

**Affiliations:** 1 Department of Adaptation Biotechnologies, Global Change Research Centre, Academy of Sciences of the Czech Republic, Drásov 470, CZ-66424 Drásov, Czech Republic; E-Mails: cerveny.j@czechglobe.cz (J.Č.); zavrel.t@czechglobe.cz (T.Z.); 2 Institute of Plant Physiology, Russian Academy of Sciences, Botanicheskaya Street 35, 127276 Moscow, Russia; E-Mail: losda@ippras.ru

**Keywords:** photosynthesis, pigments, ultrastructure, heat stress proteins, photobioreactor, cyanobacteria

## Abstract

*Synechocystis* sp. PCC 6803 is a widely used model cyanobacterium for studying responses and acclimation to different abiotic stresses. Changes in transcriptome, proteome, lipidome, and photosynthesis in response to short term heat stress are well studied in this organism, and histidine kinase 34 (Hik34) is shown to play an important role in mediating such response. Corresponding data on long term responses, however, are fragmentary and vary depending on parameters of experiments and methods of data collection, and thus are hard to compare. In order to elucidate how the early stress responses help cells to sustain long-term heat stress, as well as the role of Hik34 in prolonged acclimation, we examined the resistance to long-term heat stress of wild-type and ΔHik34 mutant of *Synechocystis.* In this work, we were able to precisely control the long term experimental conditions by cultivating *Synechocystis* in automated photobioreactors, measuring selected physiological parameters within a time range of minutes. In addition, morphological and ultrastructural changes in cells were analyzed and western blotting of individual proteins was used to study the heat stress-affected protein expression. We have shown that the majority of wild type cell population was able to recover after 24 h of cultivation at 44 °C. In contrast, while ΔHik34 mutant cells were resistant to heat stress within its first hours, they could not recover after 24 h long high temperature treatment. We demonstrated that the early induction of HspA expression and maintenance of high amount of other HSPs throughout the heat incubation is critical for successful adaptation to long-term stress. In addition, it appears that histidine kinase Hik34 is an essential component for the long term high temperature resistance.

## 1. Introduction

Cyanobacterium *Synechocystis* sp. PCC 6803 (hereafter *Synechocystis*) is a photosynthetic unicellular prokaryotic organism, widely used for studying responses and acclimation to different types of abiotic stress. Optimal growth temperature for *Synechocystis* is 32–38 °C [1]. Changes of transcriptome and proteome of *Synechocystis* [2,3,4,5,6], as well as acclimation of photosystems [7,8], cytoplasmic and thylakoid membranes [9,10,11] in response to short-term heat stress (from minutes to few hours at 43 °C and higher) are well studied.

However, the data on long term (24 h) HSPs dynamics and physiological responses, such as growth rate, intensity of respiration and photosynthesis, as well as changes in pigment content are fragmentary and not conclusive due to varying experimental conditions and incomparable methods of data collection. For example, *Synechocystis* is able to grow at 43 °C for 4 days, but then still dies [6]; at 45 °C it cannot grow at all [10]. The data on lethal temperature for *Synechocystis* differ: death occurs at temperatures 48–54 °C [2,10,12,13,14,15], wherein at 48 °C the cells must be incubated for 1–3 h [1,15], and at 54 °C—just for 5 min [10]. Apparently, the damage caused by high temperature depends on many factors, which differ in these experiments: light intensity, CO_2_ content, the growth stage, or the growth temperature before heat stress. Sheng *et al.* [16] studied the growth of *Synechocystis* sp. PCC 6803 in a 16 L flat photobioreactor at 44 °C for 4 days and the subsequent recovery at 33 °C for 10 days. At 44 °C, the growth slows down and the productivity of culture biomass is reduced, as well as the rate of absorption of nutrients. The rate of synthesis of fatty acids is also reduced together with a decrease in the unsaturation index and an increase in content of saturated palmitic acid. After 3-days incubation at 44 °C, the culture becomes bleached, but when the temperature is lowered back to 33 °C, *Synechocystis* cells are able to partially restore their original physiological features. Overall, considerable information is now available regarding the short term exposure of *Synechocystis* to high temperatures. However, the incompleteness and the existence of some discrepancies in data available on long term acclimation to heat stress, precludes a complete understanding of the heat stress response mechanisms in *Synechocystis*.

Like other organisms, *Synechocystis* responds to heat stress by induction of expression of genes for HSPs. The genome of *Synechocystis* contains several genes for HSPs, such as *htpG*, *dnaK*, *groEL*, *groES*, *hspA*, and more. An increase in the amounts of mRNAs of these genes occurs already during the first 5–30 min of heat stress, but return to a baseline levels in 1–3 h [17,18]. Amounts of the corresponding proteins are also increased within the first 20–60 min of heat stress. It is known that the level of some of HSPs remains high after 8 h of stress, despite the reduction in the amount of mRNA [19]. It is still unknown whether the amount of HSPs in *Synechocystis* goes down or remains at high level during long-term heat stress. It is also unclear whether the early cell responses help to sustain long-term heat stress or not.

Some mechanisms of regulation of heat shock response in *Synechocystis* are similar to those in other organisms. For example, alternative RNA polymerase sigma-factors SigB, SigD and SigE participate in positive regulation of several HSPs [4,6]. SigB is important for survival under short term heat stress and development of the thermotolerance [6]. Transcriptional regulator Sll1130 negatively regulates expression of *htpG*, *hspA* and some others heat-inducible genes [19]. CIRCE/HrcA system negatively regulates operons *groESL1* and *groEL2* [12]. The 5'-untranslated region of *hspA* mRNA is part of a mechanism that represses its own translation under normal conditions and derepresses it under elevated temperature or under high light [20]. Moreover, light has an influence on the extent of expression of HSP genes [21]. However, the nature of a signal, which triggers the expression of heat inducible genes, as well as the mechanism of perception of a heat stress, is still unclear [22].

In addition to common ways of regulation, the expression of many above-mentioned HSPs in *Synechocystis* is affected by histidine kinase 34 (Hik34). Hik34 functions as the sensor component of a cyanobacterial two-component signal transduction system, which is involved in generation of cellular response to abiotic stresses. Hik34 is unique among 47 histidine kinases that are encoded in the chromosome and plasmids of *Synechocystis*; no orthologs of Hik34 have been found in genomes of other bacteria. Hik34 contains only one conserved motif characteristic for histidine kinases, H1 or HisKA, which contains a phosphoacceptor and which is responsible for dimerization [23,24]. Despite the absence of any conserved ATP-binding motifs, Hik34 autophosphorylates *in vitro* at normal temperature in the presence of ATP [2]. Hik34 has no transmembrane domains and it is, probably, localized in a cytoplasm [25]. The expression of Hik34 is redox-regulated [26].

Hik34 affects the expression of sets of genes under salt and osmotic stress [25,27,28], under normal conditions and heat stress [2] and under oxidative stress [29]. In optimal and stress conditions (except oxidative stress), the common part of these sets is represented by *hspA*, *clpB1*, *dnaK2*, and *groEL2*. Hik34 is a positive regulator for these genes under salt and osmotic stress [25,27,28] and a negative regulator for them at normal growth conditions [2]. This leads to an increase in the amounts of the corresponding transcripts in the Hik34-deficient mutant, or to their decrease in Hik34 overproducing strain under normal conditions. Under short-term heat stress, the absence of Hik34 does not prevent the induction of these genes, however it affects the amplitude and dynamics of the expression. Furthermore, the ΔHik34 mutant is reported to be more tolerant to short-term heat stress (duration 1–3 h) in comparison to wild-type strain and other *hik*-deficient mutants [2,3,15,30]. The increased thermotolerance of the ΔHik34 mutant is explained by higher amounts of HSPs, which are already accumulated in cells at normal growth temperature [2]. It remains unclear, however, if Hik34 has any effect on long term stress adaptation.

In order to elucidate how the early responses help cells to sustain long-term heat stress, as well as the role of Hik34 in prolonged acclimation, we studied the resistance to long-term heat stress of wild-type and the ΔHik34 mutant of *Synechocystis* under precisely controlled conditions. To achieve the precision, we cultivated *Synechocystis* in photobioreactors, which allowed the control of wide range of light intensities and temperatures. An important advantage of photobioreactors is programmable automatic measurements of many physiological parameters, accompanied by intensive sampling.

To assess the cellular response to stress thoroughly, we analyzed physiological characteristics, such as growth and metabolic activity, as well as morphological and ultrastructural changes in cells using light, fluorescence and transmission electron microscopy. Also, the changes in amounts of some heat-shock (ClpB1, DnaK, GroEL, HspA) and other (D1, RbcL, KatG, ClpB2) proteins were monitored in both wild-type and ΔHik34 cells. The heat stress responses at the level of cell physiology, ultrastructure and heat shock protein abundance differed between wild-type and ΔHik34 mutant cells. Here, we show that the early induction of the expression of HspA and maintenance of high amount of other HSPs throughout the heat treatment (which is characteristic for the wild-type, but not to the ΔHik34 mutant cells) is necessary for successful adaptation of *Synechocystis* to long-term stress. Apparently, Hik34 is an essential component for long-term high temperature resistance.

## 2. Experimental Section

### 2.1. Strains

*Synechocystis* sp. PCC 6803 glucose tolerant strain GT-L was used as a wild-type (WT). The construction of a mutant knockout strain lacking the histidine kinase 34 gene (ΔHik34) was previously described [25]. Both strains were obtained from IPPAS collection (Institute of Plant Physiology, Moscow, Russia).

Strains were maintained on plates with solid BG-11 medium [31] buffered with 17 mM HEPES-NaOH, pH 7.5 (Sigma-Aldrich, St. Louis, MO, USA). Cells of the ΔHik34 mutant were maintained on the BG-11 medium with the addition of spectinomycin at final concentration of 30 µg mL^−1^. Pre-cultures were grown in 250 mL glass flasks in an orbital shaker at 32 °C under illumination with warm white light emitting diodes (LEDs) (SELS-3528W30, Standard Electronic Co., Ltd., Shenzhen, China) at 110 µmol (photons) m^−2^ s^−1^.

### 2.2. Experimental Setup

Cultivation of cyanobacterial strains and real-time monitoring were performed in flat-panel photobioreactors [1,32]. Illumination was provided by light panels with chessboard configuration of red and white LEDs (red: λ_max_ ≈ 627 nm, Δλ_1/2_ ≈ 20 nm, Luxeon LXHL-PD09; white: Luxeon LXHL-PW09; manufactured by Future Lighting Solutions, Montreal, QC, Canada). Photobioreactor cuvette thickness was 2.5 cm. Continuous measurements of two different optical densities and steady-state pigment fluorescence emission yield of cell cultures were performed automatically with the built-in densitometer and fluorimeter. Optical density (OD) was measured at 680 and 720 nm. Recorded fluorescence parameters were F_t_, F´_M_, and QY_PSII_, excited by 455 nm and 627 nm LEDs [32]. Dissolved oxygen and carbon dioxide were continuously measured by InPro6800 and InPro5000 electrodes (Mettler-Toledo Inc., Columbus, OH, USA). Culture temperature was monitored by InPro3253 electrode (Mettler-Toledo Inc., Columbus, OH, USA) and controlled by a Peltier cell incorporated in the instrument base. Cells were well mixed by gas bubbling complemented by rotation of magnetic stirrer bar in vertical orientation. All other accessories and properties of the experimental photobioreactors were such as described previously [33].

The experimental protocol was based on commonly used laboratory conditions. Cultures were grown at 32 °C in cuvettes with 400 mL of BG-11 medium under illumination of 80 µmol (photons) m^−2^ s^−1^ of white light supplemented with 15 μmol (photons) m^−2^·s^−1^ of red light (627 nm), aerated with 1.5% CO_2_ enriched air. The cells were cultivated at 32 °C until the middle of exponential phase previously identified at OD_680_ between 0.35 and 0.7 [1]. After that cultures were subjected to heat stress at 44 °C for 24 h, followed by recovery at 32 °C for, at least, 36 h.

### 2.3. Automatic Measurements of Respiration and Photosynthesis

Intensities of respiration and photosynthesis were measured automatically every 1.5 h. Each measurement consisted of stopping the gas flow through the bioreactor and recording a change in concentration of dissolved oxygen, caused by combined effect of photosynthesis and respiration, for 210 s. Then the light was switched off, and respiration was measured as oxygen consumption during following 210 s. After the respiration measurement, both lighting and bubbling were reintroduced, and the recordings of all parameters continued.

### 2.4. Pigment Analysis

Samples of 4.5 mL were withdrawn for pigment analysis in the following order: just before heat stress (control, time 0), after 2, 6, 16 and 24 h of heat stress, and 16 and 36 h of recovery after heat stress. Absorption spectra for wavelengths from 350 to 750 nm were measured using integrating sphere method with UV/VIS spectrophotometer (UV-2400, Shimadzu, Kyoto, Japan), with slit size adjusted to 0.5 nm. Chlorophyll *a* and carotenoid contents were measured as described previously [33].

### 2.5. FDA Activity Test 

Activity test was carried out using fluorescein diacetate (FDA; Sigma-Aldrich, St. Louis, MO, USA) [1]. FDA has been used to determine the number of active cells in different organisms including cyanobacteria [34]. FDA penetrates into cells, where it is hydrolyzed by cell enzymes, such as esterases, lipases, and proteases to form an insoluble and fluorescent compound, fluorescein, which remains in cells [35].

For activity determination, cells were pelleted from 0.1 mL samples, rinsed in PBS buffer, and stained with FDA solution at final concentration of 0.01 mg mL^−1^ at room temperature in dark for 20 min. Stained cells were analyzed on high throughput imaging flow cytometer (ImageStream® MkII, Amnis Corporation, Seattle, WA, USA) based on 10,000 cells sample statistics for each experiment time-point. Cells were gated based on intensity of their FDA fluorescence signal excited by 488 nm laser and scanned in spectral window of 505–560 nm. Positively gated cells were considered as metabolically active. The rest of the cells had the enzymatic activities below detectable level and were considered as not metabolically active, even though they might be still alive.

### 2.6. Light and Fluorescent Microscopy

Morphology of strains was examined by light microscopy (Axio Imager A1; Carl Zeiss, Jena, Germany). Inclusion bodies were stained with Nile Red at final concentration of 10 μg mL^−1^ (Sigma-Aldrich, St. Louis, MO, USA) and observed with 65 HE filter set (EX BP 475/30, BS FT 495, EM BP 550/100).

### 2.7. Electron Microscopy

For electron microscopy analysis, cells were grown in glass vessels with 250 mL of BG-11 medium in temperature-controlled water bath under continuous illumination of 110 µmol (photons) m^−2^ s^−1^ with white luminescent lamps and aerated with sterile air-gas mixture that contained 1.5%–2% of CO_2_. The experimental design was the same as described above. Samples were withdrawn just before heat stress (control, time 0) and after 2 and 24 h of heat stress. Cells were collected from 25 mL of culture by centrifugation at room temperature (4500 *g*, 5 min), fixed with 4% paraformaldehyde in 0.1 M phosphate buffer (pH 7.2) at 4 °C for 24–48 h and post-fixed with 1% OsO_4_ at room temperature for 1 h. After dehydration in ethanol and acetone, samples were embedded in Epon resin (Sigma-Aldrich, St. Louis, MO, USA). Cell sections were contrasted with uranyl acetate and then with lead citrate according to Reynolds [36], and analyzed with transmission electron microscope (Libra-120, Carl Zeiss, Jena, Germany).

### 2.8. Protein Isolation and Immunoblotting

For immunoblotting analysis, samples were withdrawn in following order: just before heat stress (control, time 0); when temperature reached 43 °C (0.3 h); than after 1.5, 2.5, 16.5 and 24 h of heat stress and 12.5 or 16.5 and 39 h of recovery. Cells (20 mL of culture) were collected by centrifugation (4100 *g*, 3 min at 4 °C) and frozen at −80 °C. Pellets were resuspended in 0.8 mL of protein extraction buffer (100 mM Tris-HCl (pH 8); 10 mM EDTA; 10 mM dithiothreitol (DTT); 5 mM aminocaproic acid; 1mM benzamidine-HCl; 1 mM phenylmethylsulfonyl fluoride (PMSF)) and disrupted with sea sand by shaking in laboratory mixer (Silamat S6; Ivoclar Vivadent AG, Schaan, Liechtenstein) for 2 min. Then SDS was added to homogenate to the final concentration of 2%, and samples were incubated at room temperature overnight for protein extraction. After that the samples were centrifuged (24,400 *g*, 10 min, room temperature), and proteins from the supernatants were precipitated with 80% (v/v) acetone at −20 °C overnight. After centrifugation, supernatants were discarded; the pellets were air dried and stored at −80 °C until analysis.

The protein pellets were dissolved in 50–150 µL of resuspension buffer (100 mM Tris-HCl (pH 8); 2% SDS). Protein concentrations were determined with the BCA protein assay (Bio-Rad, Hercules, CA, USA). Samples (10 µg) were resolved in 12.5% polyacrylamide mini gels (0.75 mm thick) by SDS-PAGE at 200 V in a MiniProtean III (Bio-Rad, Hercules, CA, USA). Proteins were either stained with CBB G-250 or transferred onto 45 µm nitrocellulose membrane (Hybond-C Extra, GE Healthcare Life Sciences) in a Trans-Blot SD Electrophoretic Semi-Dry Transfer Cell (Bio-Rad, Hercules, CA, USA) at 1 mA cm^−2^ for 2 h at room temperature. Nitrocellulose membranes were blocked overnight at +4 °C in TBSTG buffer (10 mM Tris-HCl (pH7.6); 150 mM NaCl; 0.05% (v/v) Tween-20; 1% cold-water fish gelatin) and treated with appropriate amount (as recommended by the manufacturer) of primary rabbit antisera (see the list of used antibodies in Table S1) for 2–3 h at room temperature. Proteins’ bands were visualized with secondary antibodies coupled with horseradish peroxidase.

## 3. Results

### 3.1. Physiological Changes in Wild-Type and ΔHik34 Mutant Strains of Synechocystis Subjected to Heat Stress for 24 h

**Figure 1:**
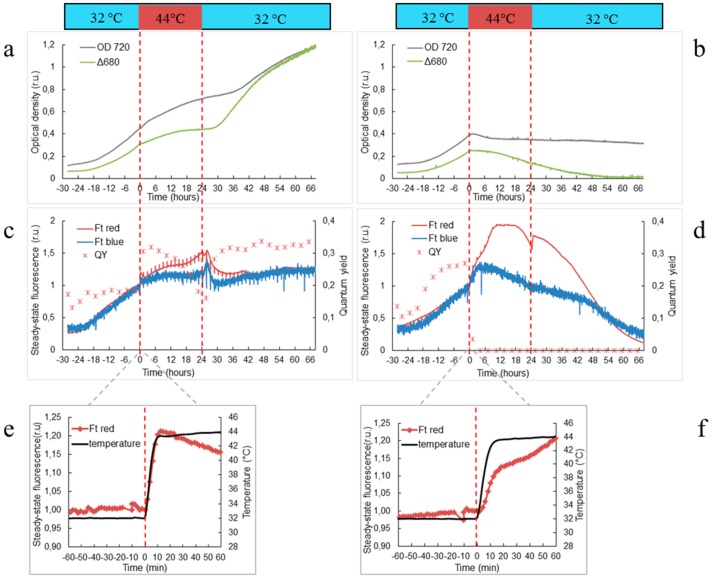
Growth of wild-type (**a,** **c,** **e**) and ΔHik34 mutant (**b,** **d,** **f**) strains of *Synechocystis* estimated by changes in optical density at 720 nm and by difference of optical densities Δ680 = OD_680_ − OD_720_ (**a,** **b**); steady state fluorescence excited by red (Ft_red_) or blue (Ft_blue_) lights (**c,** **d**); steady state Ft_red_ response during first hour of transition from 32 °C to 44 °C (**e,** **f**). Dashed lines mark time of heat shock treatment. Time zero marks the temperature shift from 32 °C to 44 °C. Cultures were growing 28 h at 32 °C before the heat shock. The graphs represent typical values from 6 and 4 independent experiments for WT and ΔHik34 mutant, respectively.

**Figure 2:**
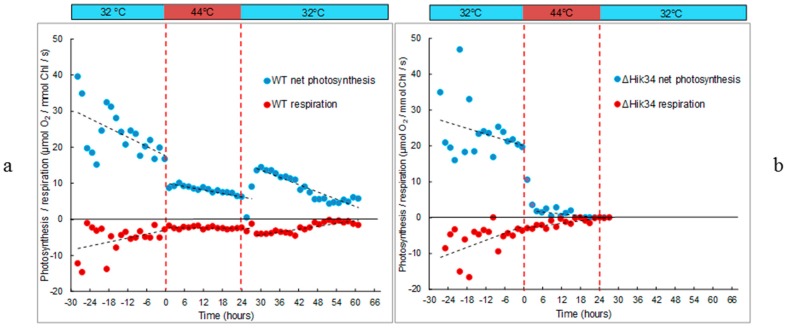
Respiration and gross photosynthesis activities of *Synechocystis* WT (**a**) and ΔHik34 mutant (**b**) measured as oxygen consumption and evolution, respectively. Dashed lines mark the time of heat shock. The results represent typical values of 4 independent experiments for both WT and ΔHik34 mutant.

Cultures were grown and monitored in flat-panel photobioreactors, which ensured precise control of cultivation conditions and allowed automatic measurements of selected physiological parameters within a time range of minutes. Using photobioreactors, we were able to monitor heat stress effects on growth rate, pigment content as well as activity of photosynthesis and respiration in *Synechocystis* wild-type and ∆Hik34 mutant cells (Figure 1 and Figure 2). We assessed the culture growth by measuring changes in OD_720_ and by measuring the difference in optical densities Δ_680_ = OD_680_ − OD_720_. It is shown that OD_720_ is approximation of biomass concentration, and Δ_680_ is proportional to the chlorophyll concentration [1,32].

Under optimal temperature of 32 °C both wild-type and ∆Hik34 mutant strains grew with the same rates with doubling times of ca. 11–12 h. However, their response to heat stress was dramatically different.

When temperature reached 44 °C, growth rate of WT slowed down immediately. It was constantly decelerating and almost ceased at the end of 24 h period of a heat shock (Figure 1a). Cell count did not change significantly during the heat shock treatment (Figure S1). After the temperature returned back to 32 °C, the growth significantly increased: after 6 h, when measured by Δ_680_, or after 11 h, when measured by OD_720_ (Figure 1a). Although the cell count didn’t rise significantly within 16 h of recovery, it was evident that cell division was resumed within the next 24 h (Figure S1).

∆Hik34 mutant stopped growing already after 1 h of heat stress (Figure 1b). After a decrease during the first 6 h, OD_720_ remained stable, while Δ_680_ was decreasing constantly. Number of cells didn’t change significantly during heat shock treatment similarly to WT (Figure S1). After temperature returned back to 32 °C, ∆Hik34 cells did not resume growth: OD_720_ remained stable and Δ_680_ continued decreasing (Figure 1b) and cell count didn’t change (Figure S1).

Significant difference in reaction to heat stress between WT and ∆Hik34 mutant was observed also in steady state fluorescence (Ft) dynamics (Figure 1c,d). The very first visible cell reaction to changes in the ambient temperature both in wild-type and mutant cells was revealed by significant increase in Ft, especially, excited by red light (Ft_red_) (Figure 1e,f). This shift occurred quickly within 10–15 min simultaneously with temperature rise from 32 °C to 44 °C.

In WT cells, Ft_red_ decreased at the beginning of heat stress, but it started to rise again after 2 h (Figure 1c). The trend of Ft_blue_ was similar, but the changes were less pronounced. During first 13 h of heat stress QY_PSII_ values were even higher than before heat stress, but after 13 h they started to decrease. After the temperature returned back to 32 °C, Ft_red_ and Ft_blue_ continued to increase for 2 h and then declined. Thereafter Ft_red_ stabilized, while Ft_blue_ was increasing until the end of the experiment.

In the ∆Hik34 mutant cells, the Ft_red_ parameter continued to grow after the first shift, and it almost doubled after 10 h of heat stress. By contrast, in wild-type cells, an increase in Ft_red_ was less than 50% (Figure 1c,d). Ft_red_ was stable for 8 h, and then it started to decline. Ft_blue_ achieved its maximum after 3–5 h of heat stress and then declined. QY_PSII_ dropped to zero after 4 h of heat stress. After the temperature returned back to 32 °C, both Ft_red_ and Ft_blue_ continued to decrease, while a QY_PSII_ remained at zero level.

Changes in photosynthetic and respiratory activity in WT and ∆Hik34 mutant cells are shown in Figure 2. Significant decrease in photosynthetic activity was evident after 1 h of heat stress in both strains (Figure 2). After that, in WT cells it slowly declined throughout the heat stress treatment (Figure 2a). Under high temperature, the intensity of respiration was also lower than at 32 °C, and it remained stable throughout the whole heat incubation. After the temperature shifted back to 32 °C, photosynthetic activity was first inhibited, but then restored in 3 h: the activity was higher than it was under stress, but lower than at initial 32 °C. The photosynthetic activity further decreased and stabilized after 26 h of recovery. Interestingly, the higher photosynthetic activity was accompanied by higher respiratory activity, which also started to decrease after 18 h at 32 °C and stabilized at low level after 26 h.

In ∆Hik34 mutant, after the initial decline, only a trace photosynthetic activity was measured after 5 h, and no activity after 16 h of heat stress treatment (Figure 2b). Respiration activity also decreased to a trace level in 16 h of heat stress. No recovery in photosynthetic or respiratory activity was observed after temperature returned back to 32 °C.

**Figure 3:**
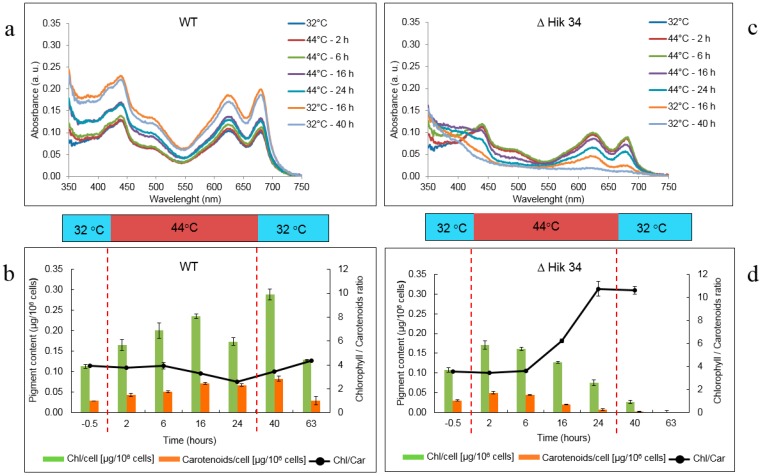
Pigment content dynamics in WT (**a, b**) and ΔHik34 mutant (**c, d**) cells during heat stress and recovery. a, c—Absorption spectra normalized to zero at 750 nm. b, d—cellular chlorophyll *a* and carotenoid content. Dashed lines mark time of heat shock treatment. The results represent a typical data of 4 independent experiments for both WT and ΔHik34 mutant. Error bars in panels b and d indicate standard deviations from 3 technical repetitions.

Changes in pigment content are shown in Figure 3. In WT cells, absorption spectra measurements (Figure 3a) showed that the content of all pigments (chlorophyll *a*, phycobilins, carotenoids) did not change significantly at high temperature. Pigments were even accumulated, though slowly, within 16 h of heat-stress treatment. Direct measurements of cellular chlorophyll *a* and carotenoid concentrations also confirmed the pigment accumulation during first 16 h of heat stress, however their contents decreased (especially of chlorophyll) between 16 h and 24 h at 44 °C (Figure 3b). A decrease in chlorophyll/carotenoids ratio from 3.9 to 2.6 was observed between 6 h and 24 h of heat stress. Pigment content was restored during recovery at 32 °C (Figure 3a,b): cellular chlorophyll and carotenoid concentrations, as well as chlorophyll/carotenoids ratio, increased significantly in 16 h and returned back to the original (control) values in 39 h.

In ∆Hik34 mutant, the content of all pigments started to decrease after 6 h of heat stress (Figure 3c,d). Based on measured spectra, carotenoids and chlorophyll were destroyed more rapidly than phycobilins. The culture of the ΔHik34 mutant changed its color accordingly: first—to bluish and then—to pale. Also UV absorbance (which may point to the presence of scytonemin) increased significantly. No recovery, in terms of changes in pigment content, was observed after temperature returned back to 32 °C.

**Figure 4:**
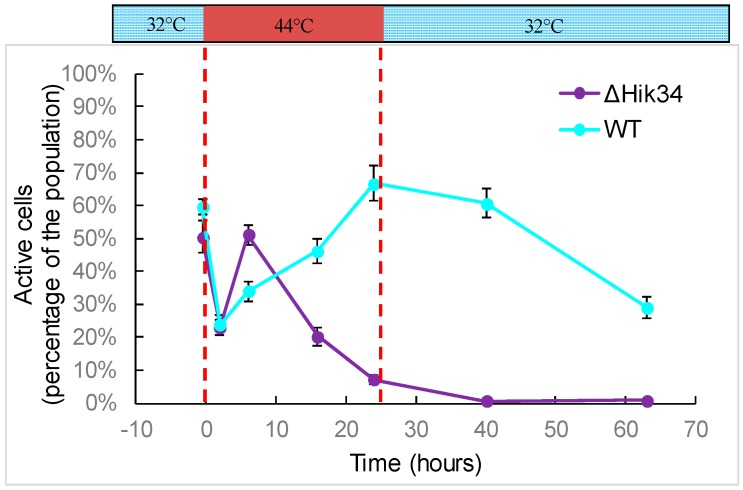
Analysis of cell activity. Dashed lines mark time of heat shock. Results represent a typical data of two independent experiments. Error bars indicate standard deviations from three technical repetitions.

FDA staining revealed that the activity in WT cells was significantly impaired during first 2 h of heat stress and then recovered. The number of active cells increased continuously during high temperature treatment (Figure 4). After temperature returned back to 32 °C, the activity continuously decreased (Figure 4).

In ΔHik34 mutant cells, first reaction to heat stress was similar to wild-type cells: at the beginning, the amount of active cells decreased and then recovered, even faster than in wild-type (Figure 4). However, after 6 h of heat stress, the number of active cells started to decrease continuously, with no recovery at 32 °C.

In contrast to wild-type, the ΔHik34 cells were unable to tolerate heat stress: their growth ceased, photosynthetic and respiratory activity dropped to zero, all pigments were destroyed (cells were bleached), and the cellular activity disappeared implying the ultimate cell death.

### 3.2. Changes in Morphology and Ultrastructure of Wild-Type and ΔHik34 Cells During Heat Stress.

**Figure 5:**
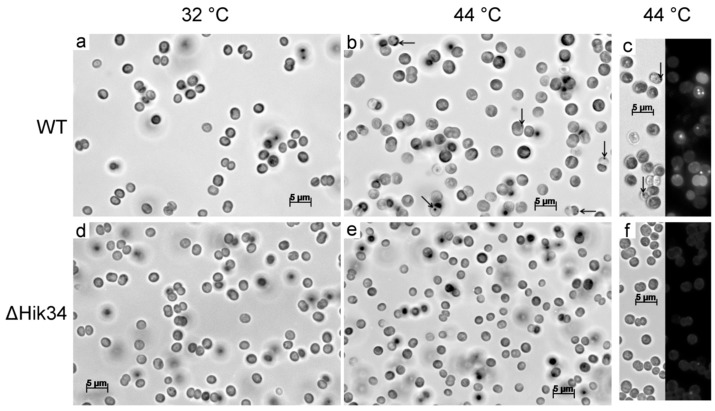
Light and fluorescent microscopy pictures of wild-type (**a**, **b**, **c**) and ΔHik34 mutant (**d**, **e**, **f**) cells. Cells were grown 24 h at 32 °C (**a**, **d**) and thereafter incubated 24 h at 44 °C (**b**, **c**, **e**, **f**). Images of cells stained with Nile Red (**c**, **f**) were received from two channels—bright field (left panel) and fluorescence (right panel). Arrows point to unidentified inclusions. Cells were analyzed in 3 independent experiments: typical microscopic photos of cells are shown.

Under heat stress, the wild-type cells became larger in size (Figure S2a), and their granularity, as measured by side scattering, increased (Figure S2b). Microscopic observations of WT cells also confirmed the increase in cell size and appearance of unidentified granules (Figure 5a,b). Also, WT cells contained granules which were stained with Nile Red (Figure 5c).

In contrast to wild-type, ΔHik34 cells did not change their size or granularity, and they did not form any granules during the heat stress (Figure S2, Figure 5d–f).

**Figure 6:**
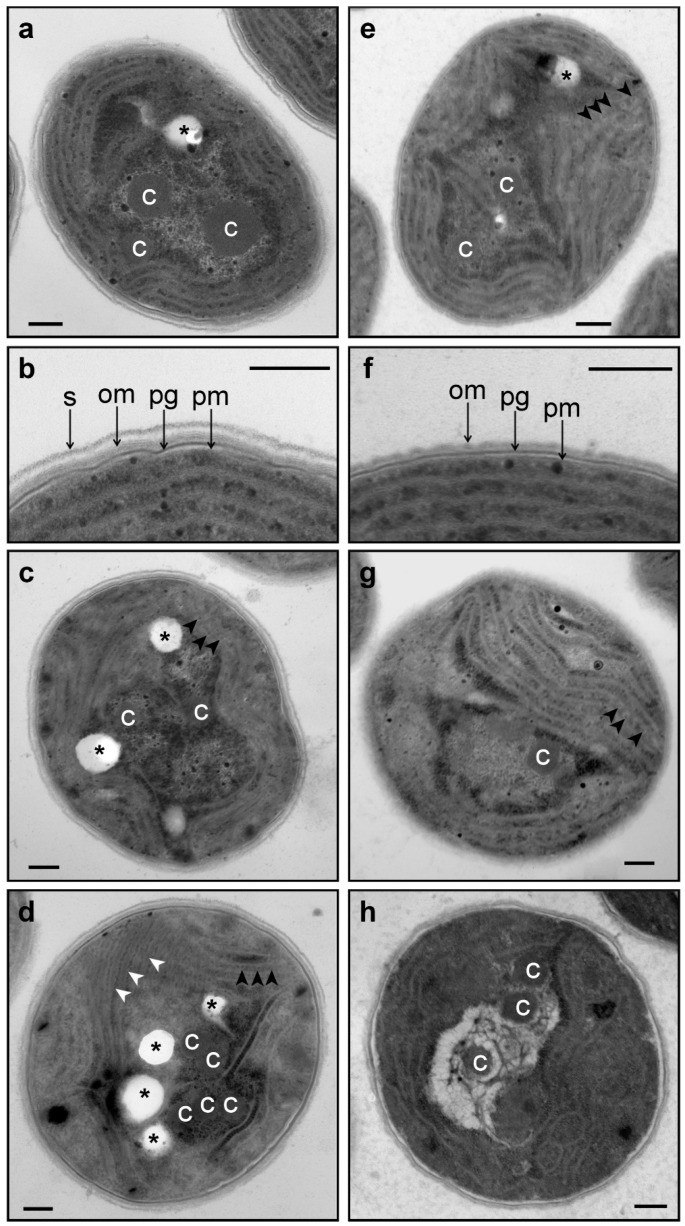
Ultrastructure of wild-type (**a**–**d**) and ΔHik34 mutant (**e**–**h**) cells grown under normal conditions (**a**, **b**, **e**, **f**) and after 2 h (**c**, **g**) and 24 h of heat stress (**d**, **h**). pm—plasma membrane; pg—peptidoglycan; om—outer membrane; s—s-layer; c—carboxysome; asterisks—electron transparent inclusions; black arrowheads show phycobilisomes; white arrowheads show thickened thylakoids with electron-dense content. All scale bars are 0.2 µm. The typical microscopic photos of cells are shown.

Ultrastructural studies of *Synechocystis* cells are shown on Figure 6. Under normal conditions, we noticed two main differences between wild-type and ΔHik34 cells (Figure 6a,b and e,f, respectively):

(1) Phycobilisomes were usually more structured and thus more apparent in ΔHik34 cells than in WT (Figure 6a,e, Figure S3a–c, S4a–c). In many ΔHik34 cells phycobilisomes formed a hatched pattern between thylakoids. Such hatched pattern was only rarely visible in WT cells under normal conditions, but it became abundant under heat stress (Figure 6c,d).
(2) The outer layer (or s-layer) was apparent in WT cells, but it was absent or significantly reduced in ΔHik34 cells (Figure 6b,f).
(3) After 2 h of heat stress, in WT cells a hatched pattern between thylakoids appeared (Figure 6с, Figure S3d–f), similar to that in ΔHik34 cells under normal conditions (Figure 6e, Figure S4a–c). This pattern was mostly formed by alternating phycobilisomes and electron-dense material between them. Some of WT cells lacked their s-layer (Figure S3d,f). The hatched pattern retained in wild-type cells during the whole heat stress period (Figure 6d, Figure S3g–i). After 24 h of heat stress, the s-layer and the outer membrane were damaged in many cells. In some cells thylakoids became thicker and filled with electron-dense material (Figure 6d, Figure S3h).

Changes were also visible in the nucleoid area. Under normal conditions or at the beginning of heat stress more electron dense parts of nucleoid (probably, more rich in ribosomes) had peripheral location. After 24 h of heat stress, electron dense regions and carboxysomes were relocated inside the nucleoid area.

For ΔHik34 mutant cells, after 2 h of heat stress (Figure 6g, Figure S4d–f) the hatched pattern was observed only in a few cells; in some cells carboxysomes looked aggregated. After 24 h of heat stress (Figure 6h, Figure S4g–i), DNA areas appeared aggregated as “bundles”. Carboxysomes lost their shape; no ribosomes were observed in the nucleoid area; no phycobilisomes were observed between thylakoids, in some cells thylakoids were curved. In addition, mutant cells, subjected to long-term heat stress, did not contain the electron transparent granules, which were always presented in wild-type cells and in mutant cells under normal conditions. According to the activity test measurements, the ΔHik34 mutant cells almost completely lost their viability after 24 h of heat stress.

### 3.3. Western-Blot Analysis of Heat Shock Induced Protein Expression

**Figure 7:**
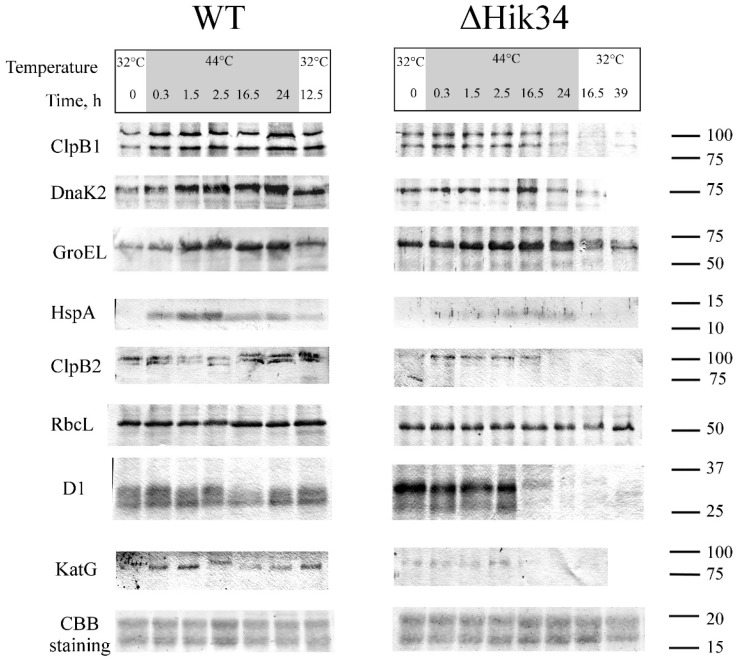
Western-blot analysis of heat-stress inducible proteins ClpB1, DnaK2, GroEL Hsp 16.6 and “constitutive” proteins ClpB2, RbcL, D1, KatG in wild-type and ΔHik34 mutant cells during 24 h of heat stress at 44 °C followed by recovery at 32°C. Last row shows parts of Coomassie R-250-stained gels. Cells were analyzed in 2 independent experiments. Typical blots are shown.

Changes in the protein levels of four heat-shock proteins, ClpB1, DnaK, GroEL, and HspA, were analyzed by western blot technique during 24 h of heat stress at 44 °C followed by 12 h of recovery at 32 °C in wild-type and 39 h of recovery in ΔHik34 mutant cells (Figure 7). Equivalent analysis has been also performed for constitutive, non-heat-inducible proteins, such as core PSII protein D1, large subunit (LS) of CO_2_ fixating Rubisco, catalase KatG, and protease ClpB2.

Before the heat treatment at 32 °C, wild-type cells displayed some basal levels of ClpB1, DnaK2, and GroEL, while HspA was not detectable. In ΔHik34 mutant, GroEL and DnaK2 were notably more abundant in comparison to WT at 32 °C. Amount of ClpB1 did not differ, and the HspA was not detected as in WT.

ClpB1 was induced in WT after 0.3 h of heat stress and remained abundant during the experiment. In ΔHik34 mutant, the amount of ClpB1 showed no significant changes up to 16.5 h of stress, and then the protein became not detectable.

DnaK2 was induced in WT after 0.3 h of heat stress as well and had highest abundance after 16.5 and 24 h. In ΔHik34 cells, the amount of DnaK2 remained stable at the beginning of heat stress and slightly increased only after 16.5 h, but then started to decrease.

Thus, the amounts and ClpB1 and DnaK2 in WT and ΔHik34 cells were similar in the first 1.5 h of the heat stress, but after that the content of these proteins was considerably higher in the WT cells.

GroEL was also induced in WT after 0.3 h of heat stress, reached its maximum already after 2.5 h, and stayed abundant until the end of a stress treatment. GroEL was the only protein, with its dynamics similar in both WT and ΔHik34, though it was more abundant in the mutant cells during first few hours of heat stress.

HspA was induced in WT after 0.3 h of heat stress with maximum abundance after 2.5 h of stress, then its amount decreased, but remained still high. Only traces of HspA were detected in ΔHik34 cells by immunoblotting between 2.5–24 h of heat stress.

A recovery at 32 °C caused a decrease in the amounts of all tested HSPs in WT, although they were higher than before treatment. In ΔHik34 cells, the amounts of ClpB1 and DnaK2 decreased already at the end of heat stress treatment. Similarly to wild-type cells, the amounts of two other heat-inducible proteins decreased at 32 °C.

While analyzing the expression of house-keeping proteins we noted that the LS of Rubisco was the most stable protein with a constant amount during the experiment in both, WT and in ΔHik34 cells. Interestingly, the amount of ClpB2 increased significantly in WT after 24 h of heat stress and remained at high level during recovery. In ΔHik34 cells, ClpB2 induction was observed after 0.3 h of heat stress, and it stayed at the same level for 16.5 h, but then disappeared.

Amount of D1 (the major protein of photosystem II reaction center) was quite stable in WT throughout heat stress treatment and recovery. In ΔHik34 cells, D1 was stable during first 2.5 h of heat stress, but then (in 16.5 h) only traces of D1 have been detected by immunoblotting. It should be noted that the D1 band of higher molecular mass was always more abundant in the mutant cells, while in WT cells these two bands displayed an equal intensity.

The amount of the catalase KatG increased in WT in first hours of heat stress, but decreased already after 2.5 h of treatment and resumed only under recovery conditions. A signal from the catalase KatG was weaker in ΔHik34 cells, if compared to WT in control conditions or during first 2.5 h of heat stress. After 16.5 h of stress this protein was almost non-detectable.

These experiments show that dynamics of both heat-inducible and constitutive, non-heat-inducible proteins differed in WT and ΔHik34 mutant cells. WT cells induced all investigated HSPs already in 0.3 h after heat stress (the minimal time, which was necessary to heat suspension from 32 °C to 43 °C) with their maximum abundance at different time points. Under recovery conditions, the amounts of all HSPs decreased in WT cells. By contrast, ΔHik34 cells already contained elevated amounts of some HSPs at normal growth conditions. However, heat stress induction of their expression was much weaker and appeared later in time, if compared to WT, with the exception of GroEL. Considering the fact that, after 16 h of heat stress, ΔHik34 cells had no photosynthetic and respiratory activities (Figure 2b), and no cellular enzymatic activity (Figure 4), the decreased amounts of HSPs may reflect the processes of cell destruction. Preservation of non-heat inducible proteins in WT under heat stress and their disappearance in ΔHik34 cells support our observation that WT cells can successfully adapt to heat stress, while ΔHik34 cells cannot.

## 4. Discussion

### 4.1. Physiological Changes in WT and ΔHik34 Mutant Strains of Synechocystis Cells Subjected to Heat Stress for 24 Hours

Growth of wild-type and ΔHik34 mutant cells at 32 °C was similar, but the heat stress responses in these two strains appeared to be rather different. In this study, we have found that wild-type cells of *Synechocystis* PCC 6803 GT-L could successfully tolerate a moderate heat stress at 44 °C for at least 24 h. The cells were able to recover from a heat stress successfully and to resume growth already after 16 h. Our data are consistent with the previous research on long-term tolerance of *Synechocystis* to similar range of temperature [6,10,16]. By contrast, the *Synechocystis* ΔHik34 mutant was able to resist heat stress only during first 1–2 h. After that mutant cells underwent irreversible changes leading to cell death. As previously reported, ΔHik34 mutant showed higher heat stress tolerance in time scale of hours [2,15,30]. Such thermotolerance of the ΔHik34 mutant was explained by higher amounts of HSPs, which were already accumulated by cells at normal growth temperature [2]. However, mutation of Hik34 has an ambiguous effect on thermotolerance of *Synechocystis*: unlike WT, the ΔHik34 mutant cells terminate growth after 10 days at 38 °C [30], whereas overexpression of Hik34 ensures an elevated thermotolerance at 48 °C, even higher than that in ΔHik34 [15].

First visible reaction of both wild-type and ΔHik34 cells to heat stress could be marked by a quick increase in Ft_red_ (Figure 1c,f). An increase in steady-state fluorescence may reflect the heat-caused reduction of a plastoquinone pool, which can be sensed by redox-sensitive signal transduction system of cyanobacteria [37], which may act as one of the mechanisms of temperature sensing. Alternatively, it could reflect uncoupling of the phycobiliproteins from the photosynthetic reaction centers.

When temperature reached 44 °C, growth of WT culture, as measured by changes in OD_720_ and Δ_680_, decelerated immediately, and almost ceased within 24 h of heat-shock (Figure 1a). Cells of the ∆Hik34 mutant stopped to grow already after 1 h of heat stress (Figure 1b). While the OD_720_ of ∆Hik34 remained mostly stable during the whole experiment, Δ_680_ decreased continuously.

Optical density of culture measured at 720 nm reflects biomass concentration [1,32]. According to cell count, cell division was arrested under heat stress in both WT and ∆Hik34 (Figure S1). Thus, OD_720_ in WT could increase due to accumulation of biomass per cell, rather than to the cell population growth. Indeed, increase in cell size and granularity (Figure S2), as well as the accumulation of some storage products was observed in WT (Figure 5). As expected from OD_720_ dynamics, cell size of ∆Hik34 and its intracellular granularity did not change (Figure S2) and no granules were observed under the microscope (Figure 5).

Consistent with Δ_680_ dynamics, pigment synthesis was not drastically impaired by heat stress in WT, though chlorophyll/carotenoids ratio shifted to carotenoid accumulation (Figure 3a,b). Such shift is considered as a common response to stressful conditions [38]. In ∆Hik34 cells, a destruction of pigments started after 6 h of heat stress (Figure 3c,d). Carotenoids and chlorophyll were destroyed more rapidly than phycobilins. In contrast to WT, chlorophyll/carotenoids ratio increased in ∆Hik34 during heat stress showing that carotenoids were even less stable than chlorophyll.

Both photosynthetic and respiratory activities decreased in WT under heat-stress after 1 h at 44 °C (Figure 2a). Rapid decline in photosynthetic activity may be caused by limitation on the site of photosynthetic apparatus. Photosynthesis during heat stress has been intensively studied and membrane fluidity has been identified to play a key role for proper photosynthesis performance during heat stress [7,9]. As a tentative explanation we suggest that the membrane integrity change (as a fast process) was connected with the decreased photosynthetic performance during heat stress. High sensitivity of the oxygen evolving complex of photosystem II to heat stress is also reported [38]. Nevertheless, in our experiments cells maintained some stable level of photosynthetic activity during heat treatment (Figure 2a). Same result is also evident from the QY_PSII_ values, which were comparable to values gained at 32 °C (Figure 1c). Some level of resistance of photosynthetic apparatus to heat stress was also confirmed by the analysis of changes in pigment content (Figure 3a,b) and amount of D1 protein (Figure 7). The accumulation of biomass in stressed cells implies ongoing carbon fixation in WT cells under heat stress.

In ∆Hik34 cells, both photosynthesis and respiration activities declined after 1 h of heat stress to levels similarly to WT. However, in contrast to WT, mutant cells displayed only trace activity after 5 h, and no activity after 16 h of heat stress (Figure 2b). Impairments in photosynthetic apparatus were confirmed also by a decrease in pigment content (Figure 3c,d). Damage to photosynthetic apparatus was reflected by a significant growth of Ft_red_ and by a drop of QY_PSII_ (Figure 1d). Moreover, immunoblotting revealed a decrease in a D1 amount in 2.5 h after heat stress followed by its complete disappearance. High Ft_red_ values may be explained by fluorescence of uncoupled phycobilisomes that are accumulated due to a decrease in the amount of functional PSII particles. The latter is reflected by a decrease in chlorophyll and D1 content [39,40].

Determination of cell activity with FDA (Figure 4) showed that the number of active cells increased in WT and decreased in the ∆Hik34 mutant cells under heat stress. The activity of esterases, proteases, and lipases, revealed by FDA, are necessary for successful acclimation to heat stress, since cells undergo significant changes in lipid and protein composition in the membranes to maintain membrane integrity and fluidity [41]. Successful acclimation of WT cells to heat stress was confirmed by rapid recovery of photosynthetic activity, pigment synthesis, and cell division after a backward shift in temperature. The ∆Hik34 mutant lost the ability to acclimate to long term heat stress conditions. Therefore a number of active cells continuously decreased up to zero, reflecting a complete loss of viability.

The estimation of photosynthetic activity surprisingly revealed that the return from 44 °C back to 32 °C was also stressful to WT cells: photosynthesis activity dropped to zero in 1 h, Ft_red_ and Ft_blue_ achieved their maxima reflecting severe disturbances in photosynthetic apparatus. It is possible that heat-acclimated cells perceived a transition from 44 °C to 32 °C as a cold stress. Photosynthetic activity, however, was restored in 2.5 h after a return of cells from 44 °C to 32 °C. Cell division was resumed only after a long lag phase (~16 h), during which all pigments were accumulated at control levels (Figure 3a).

After about 26 h of recovery cultures of WT reached high density and their growth rate started to decrease. At that time, the rates of photosynthesis and respiration decreased and stabilized at low level. The activity also decreased, which, together with photosynthesis decline may be explained by self-shading in dense culture and by other growth limitations, which occur in late linear growth phase [1,33].

### 4.2. Morphological and Ultrastructural Changes in WT and ΔHik34 Mutant Strains of Synechocystis Cells Subjected to Heat Stress for 24 Hours

Wild-type and ΔHik34 mutant cells, grown under normal conditions, did not differ under the light microscope (Figure 5a,d), although they differed in their ultrastructure. In ΔHik34 cells, phycobilisomes formed a hatched pattern between thylakoids (Figure 6e, Figure S4a,b). Such pattern was only rarely seen in WT cells under normal conditions. In *Synechocystis*, phycobilisomes are usually difficult to detect in TEM images, but they become visible when the angle of a section is parallel to the larger side of a phycobilisome [42]. Also the mutant cells had no s-layer, which was apparent in WT cells (Figure 6f and Figure 6b, respectively). According to the recent data, s-layer of *Synechocystis* consists of Sll1951 protein [43], which is shown to be absent in the soluble protein fraction of ΔHik34 cells grown at normal or high temperatures [44]. It still remains unclear how the S-protein is related to Hik34, and why it is absent (or diminished) in the ΔHik34 mutant cells.

Under heat stress, the differences between WT and ΔHik34 became apparent even under light microscope. While ΔHik34 mutant cells did not change in comparison with those under normal growth conditions (Figure 5e), WT cells increased in size (Figure 5b) and in some of them various granules appeared (Figure 5b,c). Some of the granules were stained with Nile Red and, thus, may contain polyhydroxybutyrate (Figure 5c).

Ultrastructural study revealed that under heat stress WT cells preserved their integrity and the arrangement of thylakoids, between which a hatched pattern appeared (Figure 6c,d, Figure S3d–i). Hatched pattern was formed by arranged alternating phycobilisomes and by an electron-dense material that fills a space between them. In some cells thylakoids became thicker and were filled with electron-dense material (Figure 6d, Figure S3h). Damage was only noticeable by disappearance or disruption of s-layer and sometimes also the outer membrane, especially, after 24 h of stress treatment.

Unlike WT, ΔHik34 cells demonstrated destructive changes in their ultrastructure after 24 h of heat stress: ribosomes disappeared in the nucleoid area, DNA formed bundles, carboxysomes aggregated, and thylakoids curved. According to physiological data described above, mutant cells were not viable after 24 h of heat stress.

### 4.3. Protein Analysis

The dynamics of both heat-inducible and constitutive proteins in ΔHik34 mutant significantly differed from that in WT cells. In mutant cells grown at normal temperature, the heat-shock proteins GroEL and DnaK2 were notably more abundant than in WT, which is consistent with previous observation [3]. The amount of ClpB1 in the mutant cells did not significantly differ from WT, and HspA was not detected in any of the strains. Taking into account that western blotting allows to determine only significant quantitative differences, this observation is consistent with previous reports that show 1.5–1.8-fold difference in the amount of ClpB1 and 1.6-fold difference in the amount of HspA between WT and mutant cells, where the signal from HspA in WT cells is barely distinguishable [2,19].

Under heat stress, only GroEL displayed similar dynamics in both strains, though its initial amount was higher in the mutant. This means that GroEL is less dependent on Hik34 under heat stress than other HSPs, and that it might be controlled by other regulators.

Amounts of ClpB1 and DnaK2 were similar in WT and ΔHik34 during the first 1.5 h of heat stress, but then these amounts were considerably higher in WT cells. In WT, ClpB1 and DnaK2 increased during 24 h of heat stress in contrast to ΔHik34, in which no increase was observed. This means that Hik34 is necessary for the early induction of expression of these proteins and for the maintenance of their expression under heat stress.

The most notable was the quick appearance of HspA and its high amount under heat treatment in WT cells. By contrast, ΔHik34 displayed only traces of HspA between 2.5 and 24 h of heat treatment. Therefore, under our experimental conditions, HspA was controlled by the unreplaceable regulator, Hik34. This observation contradicts with a previous report [3], where soluble proteins of WT and ΔHik34 mutant were compared. Here we compared proteins from a whole cell extracts that contained both soluble and membrane proteins. The growth phase of cells also differed: we used cells in exponential growth phase, whereas other experiments have been performed with cells taken from linear growth phase [3,44].

Deletion of the gene for Hik34 has an ambiguous effect on the induction of HSPs both at the transcriptional and translation level [2,3,44]. The induction of many HSP genes is retarded in ΔHik34 but lasted longer, if compared to WT cells [2]. In the above experiments, the amounts of HSPs were compared only at one point of the heat stress (namely, in 60 min): some HSPs were more induced in WT, and some—in ΔHik34 cells [44]. This implies that the expression of HSP genes is controlled by several different regulators.

We observed that, in WT cells, four HSPs were induced together with heating. It might be that the elevated amounts of HSPs produced in the ΔHik34 mutant cells under normal conditions were sufficient to protect cells against short-term heat stress, but not enough against long-term heat stress, when damage and impairments were accumulating. Such impaired accumulation of HspA and, to a lesser extent, of DnaK2 and ClpB1 in ΔHik34 may explain the differences in physiological responses of the mutant compared to WT cells. It can also be the reason of inability of the ΔHik34 mutant to survive a long-term heat treatment.

A lack of HspA in the ΔHik34 mutant under heat stress may also explain rather fast impairment of photosynthesis and pigment bleaching. HspA is shown to protect membrane proteins of photosystems as well as soluble proteins of phycobilisomes from the oxidative damage. Similarly to the ΔHik34 mutant under heat stress, the ΔHspA cells under oxidative stress are characterized by the reduction of carotenoids and chlorophyll, which is more intensive than reduction of phycocyanins [45]. Since heat stress is always accompanied by oxidative stress [38], the mechanisms of inhibition of photosynthetic machinery might be similar under these two stresses. Early studies demonstrate that ΔHspA mutant cells decrease their growth rate significantly and reduce oxygen evolution rates by 60% after incubation at 42 °C for 20 min, compared to a 10% decrease in wild-type cells [46]. Decreased amount of DnaK2 protein can also contribute to inactivation of photosynthetic apparatus, as the photosynthetic activity is more sensitive to heat stress in the DnaK2-deficient mutant [47]. Damage to a photosynthetic machinery in ΔHik34 might result from elevated amounts of ROS [15] In addition, here we observed that KatG, which is necessary to protect cells against oxidative stress, was not detectable in the mutant cells after 16.5 h of heat stress.

It is possible that higher order in phycobilisome arrangement (revealed by hatched pattern between thylakoids), which is visible in heat stressed WT cells and in ΔHik34 cells under normal conditions, may appear due to accumulation of chaperone proteins. GroEL [48,49], HspA [9,11,50,51] and DnaK2 [47,49,52] are found to be bound to the thylakoid membranes of cyanobacteria. In our study, mutant cells had higher amounts of GroEL and DnaK proteins under normal conditions and lower amounts of HspA and DnaK under heat stress, while WT cells had elevated amounts of GroEL, DnaK and HspA proteins during all the time of heat stress. Hatched pattern was preserved in WT after 24 h of heat stress. In mutant cells it was observed only under normal conditions or in less extent after 2 h of heat stress. Apparently, time-course of chaperone distribution corresponds to time of existence of hatched pattern in wild-type and mutant cells, and thus provides the basis for a suggestion that this pattern may be formed due to accumulation of chaperones. Electron-dense deposits in the lumen of thylakoids observed in heat stressed WT cells (Figure 6d, Figure S3h) can consist of HspA according to a previous report [13].

ΔHik34 cells had also impaired structure of nucleoid after 24 h of heat stress. DNA formed aggregated fibers in mutant cells, while in wild-type cells there were no such aggregates. Similar fibrillar DNA structures are described in the ΔHspA mutant cells of *Synechocystis* under salt stress [53]. Supposedly, HspA can interact with some DNA-binding proteins under stress conditions. HspA can also stabilize nucleoid structure under heat stress in *Synechococcus* strain ECT16-1 [51]. Since ΔHik34 cells had significantly lower amounts of HspA than wild-type cells (Figure 7), the appearance of DNA aggregates may be related to a lack of HspA.

## 5. Conclusions

The data presented in this study show that the role of histidine kinase 34 in heat stress response is very dramatic, yet is not decisively defined in terms of mechanism. Although ΔHik34 mutant cells were resistant to a short-term heat stress (1–2 h), they could not survive a long-term heat stress (24 h). By contrast, WT cells were able to recover after 24 h of cultivation at 44 °C. Under heat stress, in ΔHik34 cells, the heat shock protein HspA was not induced and the amounts of other heat shock proteins ClpB1 and DnaK2 were lower than in WT.

It has been shown that Hik34 regulates ClpB1, DnaK2, GroEL, and HspA positively under salt and osmotic stress [25,27,28] and negatively under normal growth conditions [2,3]. Previously published data [2,3,44] and our present results suggest that Hik34 can also function as bidirectional regulator under heat stress. Autophosphorylation state of Hik34 depends on ambient temperature [2]. In the bacterial world, there are some examples of two-component regulatory systems, in which a sensory histidine kinase can switch from kinase to phosphatase activity depending on its autophosphorylation state. In such way it can operate as a positive or a negative regulator [54].

The existence of several response regulators that are differentially phosphorylated by Hik34 under different environmental conditions cannot be excluded. The presence of some auxiliary proteins, similar to SipA-like protein interacting with Hik33 [55], which may switch or shift the activity of histidine kinases, is also possible. Furthermore, induction of HSPs in ΔHik34 mutant under heat stress could not be explained merely by the absence of functional Hik34. Some other regulators like CIRCE/HrcA system, sigma-factors SigB, SigD, and SigE, transcriptional factor Sll1130, or yet unknown regulator(s), may participate in HSPs induction/derepression. In our experiments, these complementary mechanisms might appear in GroEL regulation, but, probably, not in DnaK2, ClpB1, and, specifically, HspA regulation. The latter is the important component of the resistance to oxidative stress, which accompanies heat stress. The mechanisms of inhibition of photosynthetic machinery might be similar under these two stresses. Thus, the ability of cells to tolerate heat stress might be related to their ability to cope with the stress-induced appearance of reactive oxygen species. In that sense, the role of Hik34 in multiple stress resistance of *Synechocystis*, as well as the physical nature of a signal perceived by this histidine kinase, should be further elucidated.

## Supplementary Materials

Supplementary materials can be accessed at: http://www.mdpi.com/2075-1729/5/1/676/s1.
