# Supplementary material for: Mechanisms of High Temperature Resistance of Synechocystis sp. PCC 6803: An Impact of Histidine Kinase 34
Source: Life (Basel). 2015 Mar 2;5(1):676–99. doi: 10.3390/life5010676 (PMC4390874; doi:10.3390/life5010676)
Supplement: Supplementary file 1 [file life-05-00676-s001.pdf]

Supplementary Materials

Materials and Methods

Cells Size and Granularity Measurements

Cell size and granularity determination was estimated by the analysis of images acquired on ImageStream® MkII (Amnis Corporation, Seattle, WA, USA) high throughput imaging flow cytometer based on 10,000 cells sample statistics for each experiment time-point.

Table S1. List of antibodies used in this study. All antibodies were raised in rabbits.

Designation	Antiserum description
ClpB1	AS08 344 (Agrisera, Sweden) raised against recombinant ClpB1 protein, derived from Slr1641 of *Synechocystis* PCC 6803 strain sequence; protein has an internal translation site.
ClpB2	AS08 355 (Agrisera, Sweden) raised against recombinant Slr0156 protein, derived from *Synechocystis* PCC 6803 strain *slr0156* sequence. This protein is annotated as ClpB1 in a data base but was originally named ClpB2 according to [1].
D1	AS10 704 (Agrisera, Sweden) raised against KLH-conjugated synthetic peptide, amino acids 234-242 of D1 protein P83755 (AtCg00020)
DnaK2	AS08 350 (Agrisera, Sweden) raised against full length recombinant DnaK2 protein derived from *Synechocystis* PCC 6803 DnaK2 protein sequence P22358
GroEL	Raised against a mixture of Hsp60s (homologous GroEL1 and Cpn60) of *Synechocystis vulcanus* [2].
HspA	AS08 286 (Agrisera, Sweden) raised against recombinant protein. *Synechocystis* PCC 6803 Hsp16.6 CI (class one) P72977
KatG	AS08 374 (Agrisera, Sweden) raised against recombinant full length KatG protein from *Synechocystis* sp. PCC 6803 (accessions P73911 and Sll1987) with six His-tag on the terminus
RbcL	AS03 037 (Agrisera, Sweden) raised against KLH-conjugated synthetic peptide conserved across all known plant, algal and (cyano)bacterial RbcL protein sequences (form I L8S8 and form II L2), including *Arabidopsis thaliana* AtCg00490, *Hordeum vulgare* P05698, *Oryza sativa* P0C510, *Chlamydomonas reinhardtii* P00877, *Synechococcus* PCC 7920 A5CKC5

Table S2. Genes regulated by histidine-kinase 34 under different abiotic stresses.

Genes that are positively regulated by Hik34 under salt stress [3,4]	Genes that are positively regulated by Hik34 under osmotic stress [5]	Genes that are negatively regulated by Hik34 under normal conditions and heat stress [6,7]
hspA	*hspA*	*hspA*
clpB1	*clpB1*	*clpB1*
dnaK2	*dnaK2*	*dnaK2*
groEL2	*groEL2*	*groEL2*
dnaJ	*groEL1*	*groEL1*
hik34	*dnaJ*	*groES*
sodB	*hik34*	*htpG*
slr1915	*sodB*	*sigB*
sigB	*slr1915*	*hliC*
pbp	*groES*	*sll1634*
slr1916	*htpG*	*hemA*
sll1884	*spkH*	*cpcC*
sll1107	*sll0846*	*nrtD*
slr0959	*slr1963*	*atpF*
sll0528	*ssl2971*	*ssl3044*
slr0095	*slr1413*	*psbD2*
slr1687	*slr0959*	*psbA3*
slr0960	*sll1884*	*sll0846*
slr1917	*slr1603*	
sll1541		
ribF		
hypA		
ssl3044		
slr1674		
ssr3188		
leuB		
sll1757		
slr1918		
sll1106		

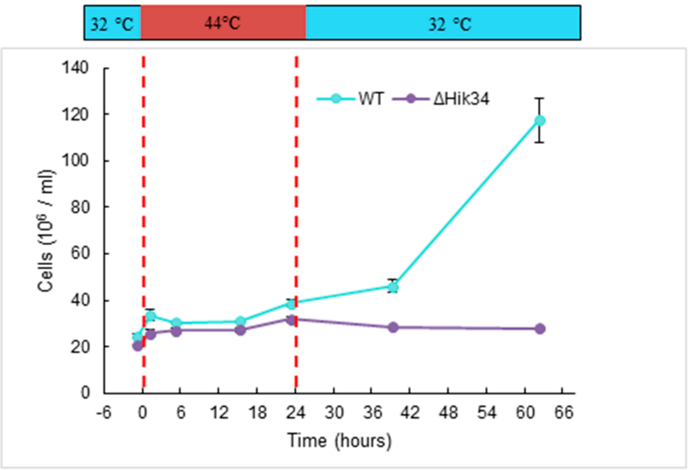

Figure S1. Number of cells per ml of culture during and after heat stress at 44 °C for 24 h. Before heat stress at 44 °C, the culture was cultivated at 32 °C for 28 h. Error bars represent standard deviation. The experiments were performed in duplicates, data from one representative experiment are shown.

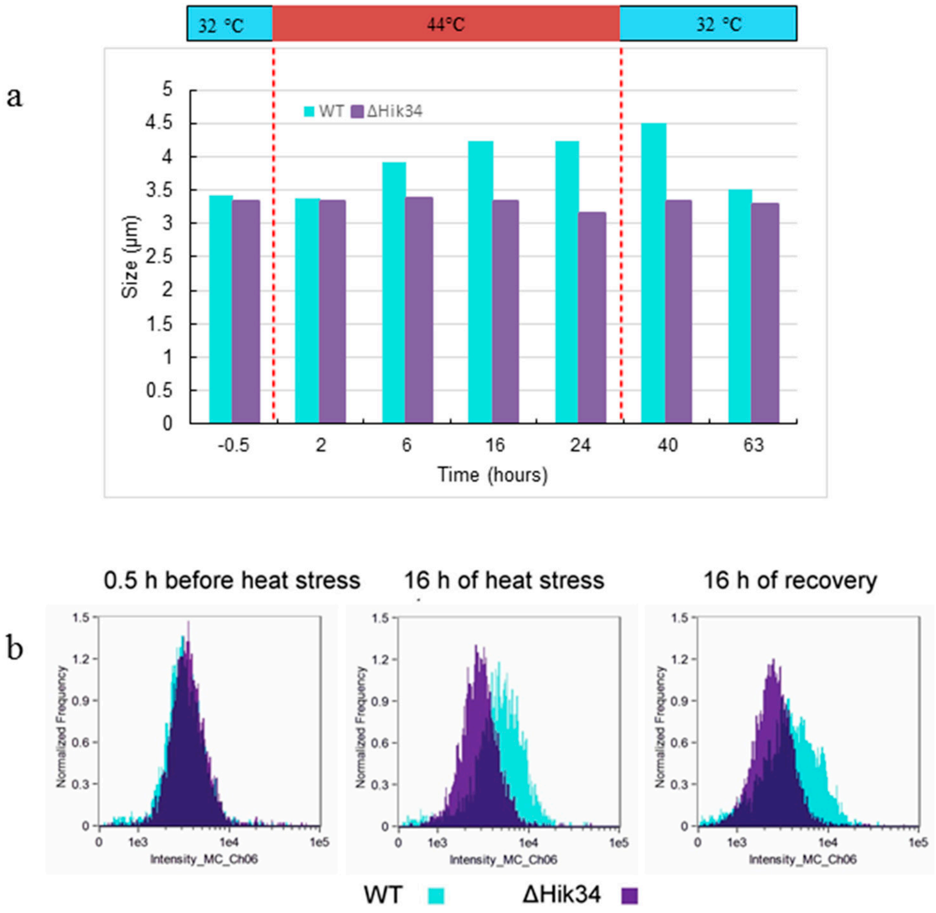

Figure S2. (**a**) Changes of cell sizes of WT and ΔHik34 mutant during the experiment. To provide better interpretation regarding all population, medians of cell size are given, as median goes toward the most abundant cell size in population. Dashed lines show time of heat shock treatment. (**b**) Side scatter distributions of WT and ΔHik34 cells under normal conditions, after 16 h of heat shock and after 16 h of recovery from 24 h of heat stress.

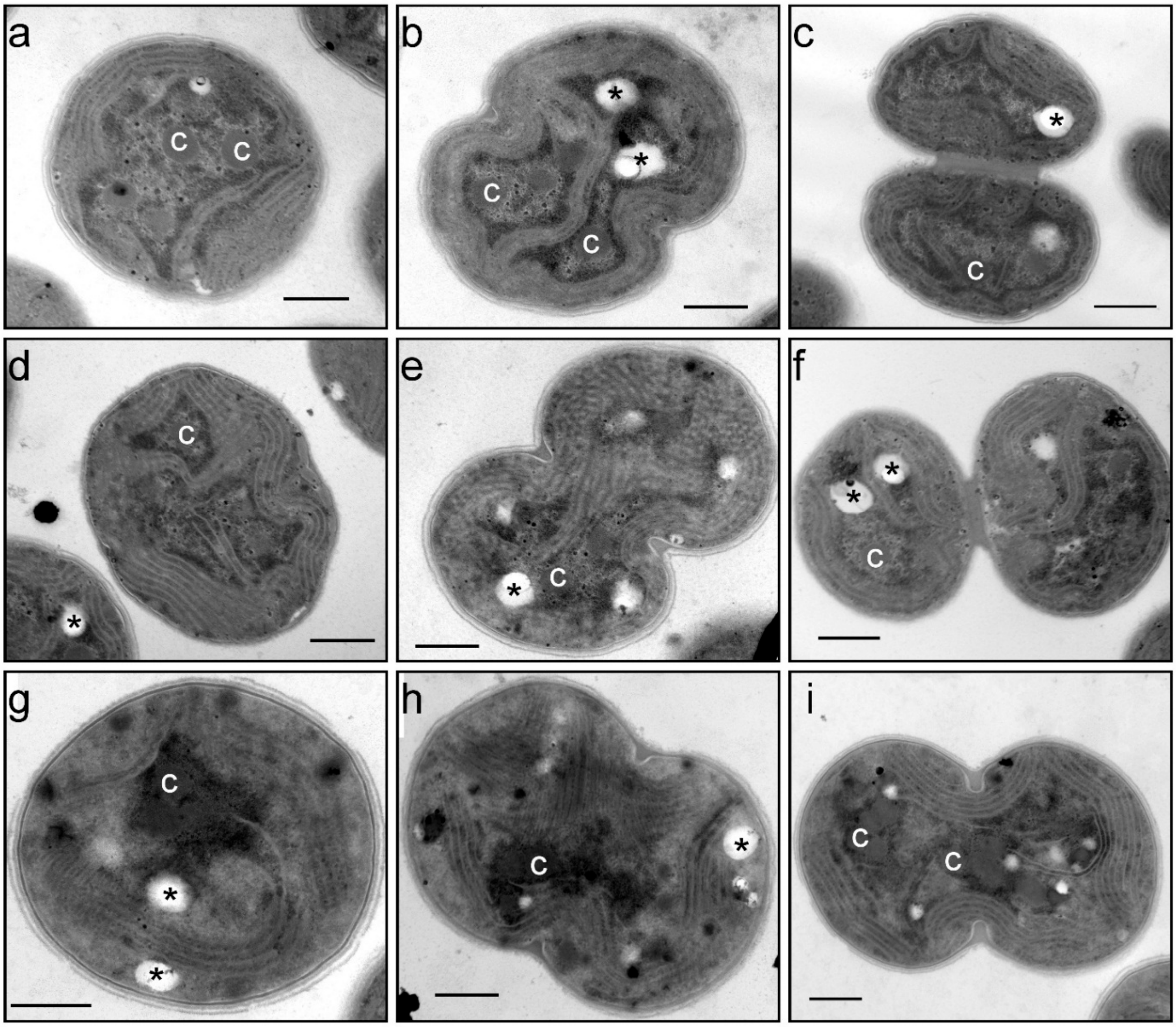

Figure S3. Ultrastructure of wild-type cells under normal (control) conditions (**a-c**); after 2 h (**d**–**f**), and after 24 h (**g**–**i**) of heat stress. c—carboxysome, asterisks—electron transparent inclusions. All scale bars correspond to 0.5 μm.

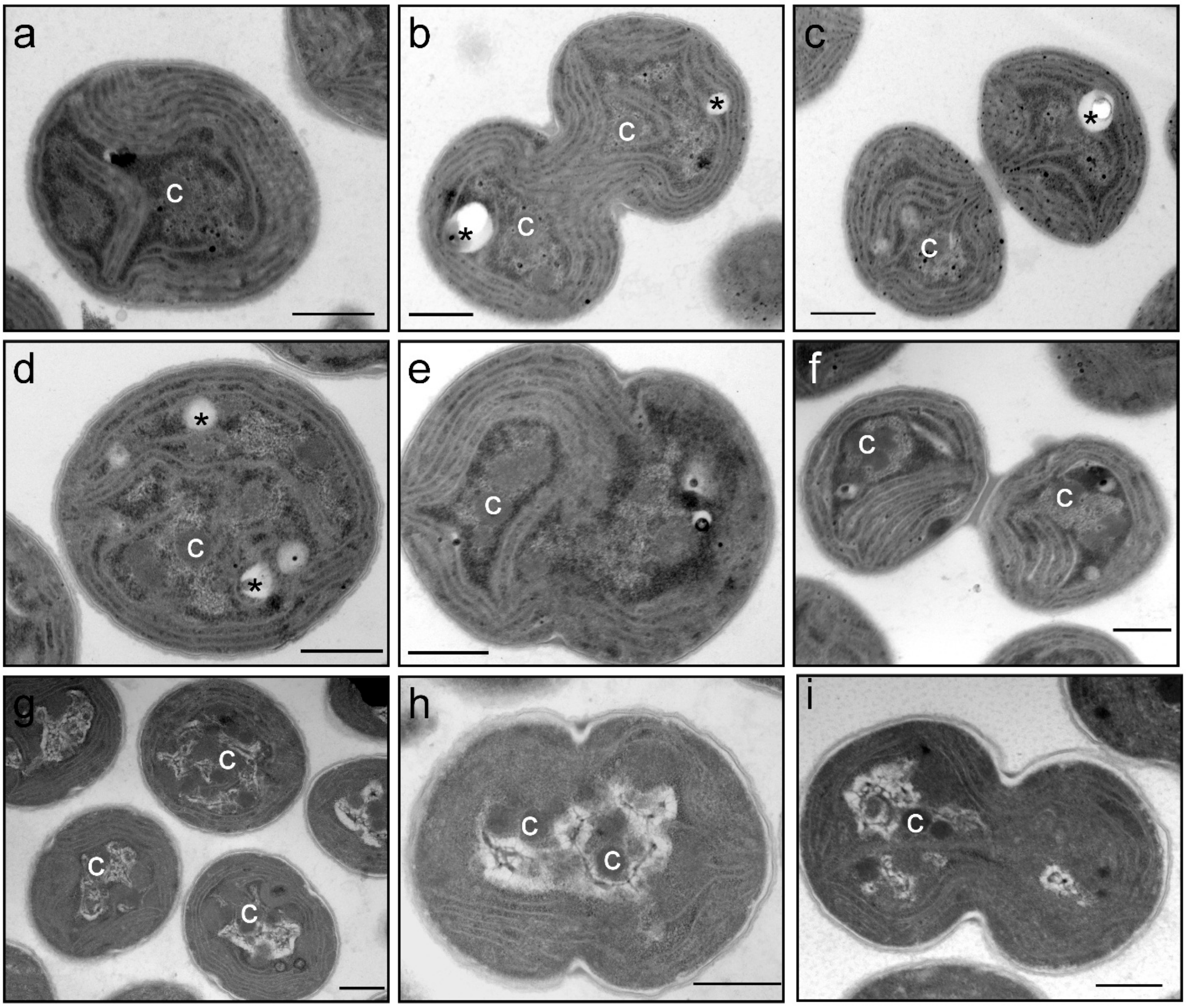

Figure S4. Ultrastructure of ΔHik34 mutant cells grown under normal (control) conditions (**a**–**c**); after 2 h (**d**–**f**), and after 24 h (**g**–**i**) of heat stress. c—carboxysome, asterisks—electron transparent inclusions. All scale bars correspond to 0.5 μm.

References

1. Giese, K.C.; Vierling E. Changes in oligomerization are essential for the chaperone activity of a small heat shock protein in vivo and in vitro. *J. Biol. Chem.* **2002,** *277*, 46310–46318.
2. Nishiyama, Y.; Los, D.A.; Murata, N. PsbU, a protein associated with photosystem II is required for the acquisition of cellular thermotolerance in *Synechococcus* species PCC 7002. *Plant Physiol.* **1999**, *120*, 301–308.
3. Marin, K.; Suzuki, I.; Yamaguchi, K.; Ribbeck, K.; Yamamoto, H.; Kanesaki, Y.; Hagemann, M.; Murata, N. Identification of histidine kinases that act as sensors in the perception of salt stress in *Synechocystis* sp. PCC 6803. *Proc. Natl. Acad. Sci. USA* **2003**, *100*, 9061–9066.
4. Shoumskaya, M.A.; Paithoonrangsarid, K.; Kanesaki, Y.; Los, D.A.; Zinchenko, V.V.; Tanticharoen, M.; Suzuki, I.; Murata, N. Identical Hik-Rre systems are involved in perception and transduction of salt signals and hyperosmotic signals but regulate the expression of individual genes to different extents in *Synechocystis*. *J. Biol. Chem.* **2005**, *280*, 21531–21538.

5. Paithoonrangsarid, K.; Shoumskaya, M.A.; Kanesaki, Y.; Satoh, S.; Tabata, S.; Los, D.A.; Zinchenko, V.V.; Hayashi, H.; Tanticharoen, M.; Suzuki, I.; *et al.* Five histidine kinases perceive osmotic stress and regulate distinct sets of genes in *Synechocystis*. *J. Biol. Chem.* **2004**, *279*, 53078–53086.
6. Suzuki, I.; Kanesaki, Y.; Hayashi, H.; Hall, J.J.; Simon, W.J.; Slabas, A.R.; Murata, N. The histidine kinase Hik34 is involved in thermotolerance by regulating the expression of heat shock genes in *Synechocystis*. *Plant Physiol.* **2005**, *138*, 1409–1421.
7. Slabas, A.R.; Suzuki, I.; Murata, N.; Simon, W.J.; Hall, J.J. Proteomic analysis of the heat shock response in *Synechocystis* PCC 6803 and a thermally tolerant knockout strain lacking the histidine kinase 34 gene. *Proteomics* **2006**, *6*, 845–864.
